# Measures for assessing practice change in medical practitioners

**DOI:** 10.1186/1748-5908-1-29

**Published:** 2006-12-06

**Authors:** Sharon Hakkennes, Sally Green

**Affiliations:** 1 School of Physiotherapy, La Trobe University, Victoria, Australia; 2 Monash Institute of Health Services Research, Monash University, Victoria, Australia

## Abstract

**Background:**

There are increasing numbers of randomised trials and systematic reviews examining the efficacy of interventions designed to bring about a change in clinical practice. The findings of this research are being used to guide strategies to increase the uptake of evidence into clinical practice. Knowledge of the outcomes measured by these trials is vital not only for the interpretation and application of the work done to date, but also to inform future research in this expanding area of endeavour and to assist in collation of results in systematic reviews and meta-analyses.

**Methods:**

The objective of this review was to identify methods used to measure change in the clinical practices of health professionals following an intervention aimed at increasing the uptake of evidence into practice. All published trials included in a recent, comprehensive Health Technology Assessment of interventions to implement clinical practice guidelines and change clinical practice (n = 228) formed the sample for this study. Using a standardised data extraction form, one reviewer (SH), extracted the relevant information from the methods and/or results sections of the trials.

**Results:**

Measures of a change of health practitioner behaviour were the most common, with 88.8% of trials using these as outcome measures. Measures that assessed change at a patient level, either actual measures of change or surrogate measures of change, were used in 28.8% and 36.7% of studies (respectively). Health practitioners' knowledge and attitudes were assessed in 22.8% of the studies and changes at an organisational level were assessed in 17.6%.

**Conclusion:**

Most trials of interventions aimed at changing clinical practice measured the effect of the intervention at the level of the practitioner, i.e. did the practitioner change what they do, or has their knowledge of and/or attitude toward that practice changed? Less than one-third of the trials measured, whether or not any change in practice, resulted in a change in the ultimate end-point of patient health status.

## Background

The healthcare professions are increasingly considering available evidence when determining best practice. Clinical practice guidelines based on the most recent and reliable evidence are designed to keep practitioners up-to-date with research and assist them in the process of implementing evidence into practice [1]. However, unless clinicians adhere to the recommendations of practice guidelines they will have little or no impact on actual clinical practice.

Each year a vast number of studies are published and aim to assess the impact of various strategies for effectively disseminating and implementing clinical practice guidelines. Numerous systematic reviews have been written that evaluate the effectiveness of these different strategies. Recently, in the most comprehensive review to date, Grimshaw et al. systematically reviewed 235 studies evaluating the effectiveness and cost of various guideline development, dissemination, and implementation strategies [2]. The majority of the studies included in this review used measures of process rather than actual patient outcomes, even though only three of the guidelines were overtly evidence-based. Although there was an improvement in process of care for the majority of comparisons reporting dichotomous data (86.6%), the authors found considerable variation in results both within and between included studies. The authors concluded that there is insufficient evidence to determine which guideline dissemination and implementation strategies are likely to be effective under different circumstances, and highlighted the need for further research.

The Cochrane Effective Practice and Organisation of Care group (EPOC) performs systematic reviews of interventions designed to improve professional practice and the delivery of health services [3]. For example, the review by O'Brien et al concluded that interactive workshops could result in moderately large changes in professional practice, while didactic lectures alone were unlikely to effect change [4]. Together, these studies and reviews are guiding the strategies that health professionals and policy makers use to facilitate the uptake of evidence into clinical practice.

Knowledge of the outcome measures employed by studies investigating the effectiveness of strategies to change practice is vital, not only for the interpretation and application of the work done to date, but also to inform future research in this expanding area of endeavour. Appropriate choice of outcome measure is important in ensuring both the internal and external validity of studies. That is, the chosen outcome measure needs to display a high level of consistency when measuring the outcome (reliability) and needs to measure what it is intended to measure (validity). If these two components are not present, the ability to reasonably interpret the data presented in the trial and to generalize the results outside of the trial is compromised.

Increasingly, specific clinical areas of health care are developing standarised core sets of outcome measures for use in research. One example of this is OMERACT (Outcome Measures in Rheumatoid Arthritis Clinical Trials) [5]. OMERACT has been described as an informal gathering of professionals interested in outcome measurement in rheumatology and aims to improve outcome measurement by gaining consensus over which measures are applicable in trials for each clinical indication[6,7]. This process not only aids clinical research by standardising methodology, but also drives further research in areas where the lack of available research means evidence-based decisions cannot be made, and assists in the synthesis of results in systematic reviews and meta-analyses.

In order to make recommendations about choice of outcome measures for evaluating the effect of implementation strategies, the measures currently used need to be described. Importantly, the level at which the effect of the intervention is being measured (patient versus health practitioner versus organisational/system) needs to be determined. The reliability and validity of these measures and methods also need to be established.

To date, no published studies have attempted to assess which outcome measures have been used to determine the effectiveness of strategies aimed at improving development, dissemination, and implementation of clinical practice guidelines.

The primary objective of this study was to identify methods that have been used to evaluate the outcome of strategies for the dissemination and implementation of guidelines.

The secondary objectives were to describe the way in which the outcome measures were applied, the time taken to collect the data for each of these measures, and, where reported in the studies, the reliability and/or validity of the methods used.

## Methods

The recent systematic review by Grimshaw et al. was used as the source of included studies for this review [2]. This review performed a comprehensive search of the literature (1966–1998) and identified 235 studies (randomised controlled trials, controlled clinical trials, controlled before and after studies and interrupted time series studies) that assessed the effectiveness of various guideline dissemination and implementation strategies.

We obtained copies of all included studies for our data extraction. Due to difficulties in obtaining the papers, studies not published in peer-review journals were excluded. To be included in the data extraction, the outcome measure(s) needed to be detailed in the methods and/or results sections of the included study.

One reviewer (SH) used a standardised data collection form to extract data from the included studies. Extracted data comprised information about the measure(s) used to assess the effectiveness of the intervention, method(s) used to collect data for the outcome measure(s), and the reliability and/or validity of the chosen outcome measure (where reported in the included study).

Outcome measures used were grouped into five distinct categories based on the following criteria:

A. Patient level

1. Measurements of actual change in health status of the patient, i.e., pain, depression, mortality, and quality of life. (A1)

2. Surrogate measures of A1, i.e., patient compliance, length of stay, and patient attitudes. (A2)

B. Health practitioner level

1. Measurements of actual change in health practice, i.e., compliance with guidelines, changes in prescribing rates. (B1)

2. Surrogate measures of B1, such as health practitioner knowledge and attitudes. (B2)

C. Organisational or Process level

Measurements of change in the health system (i.e., waiting lists), change in policy, costs, and usability and/or extent of the intervention. (C)

In instances where the outcome measure was aimed at measuring change in more than one category, it was recorded in both of the categories (e.g. a measure of number of mammograms where the intervention was targeted at changing patient and health practitioner behaviour was classified as A2 and B1). When the intervention was only targeting health practitioner behaviour, the intervention was classified only as B1.

Data were then extracted for each study, as to the number of different categories the outcome measures represented. Rather than extracting the actual number of outcome measures used, this approach was the most appropriate way to represent the results, as the objective of this study was to determine the types of outcome measures being used to assess practice change rather than the frequency of their use within studies.

For each included study, the various methods for collecting the data for the outcome measures were extracted. Each method of data collection was counted only once, even if the same method was used more than once for different outcomes in the same included study; for example, patient survey to determine health practitioner compliance and patient satisfaction questionnaire. Conversely, a study may have used more than one method to collect data for the same outcome measure (e.g. both a computerised and manual medical record audit). In this instance, both outcome measure methods were counted.

One of the authors, (SH) following a pilot of the data extraction (n = 20), identified the methods used to collect the data for the outcome measures and grouped them into nine categories:

1. Medical record audit

• Data collected through audit of the patients' chart/medical history;

2. Computerised medical record audit

• Data collected through an audit of a computerised version of the patients' chart/medical history;

3. Health practitioner survey/questionnaire/interview

• Data collected from the health care provider through surveys or questionnaires either written or verbal, and interviews either in person or by telephone;

4. Patient survey/questionnaire/interview

• Data collected from patients, families, and/or the general community through survey and questionnaires either written or verbal, and interviews either in person or by telephone;

5. Computerised database

• Data collected from centralised computer databases such as pharmacy prescription registers, medical billing information;

6. Log books/department record/register

• Data collected from organisational documents such as a register of presentations to the emergency department, log book of x-ray requests;

7. Encounter chart/request slips/diary

• Data collected from forms completed by the health practitioner, encounter forms designed for the study containing details required for the data collection, request slips (i.e., laboratory tests), and/or diary kept for the study data collection;

8. Other

• Data collected from outcome measures not included in any of the above categories, such as clinical examination, results of blood tests, video/audio taping of consultations; and

9. Unclear

• The exact method of data collection could not be established.

Following data extraction, data were entered into a database and descriptive statistics used to describe the proportions of studies using various levels of outcome.

## Results

Of the 235 studies included in the systematic review by Grimshaw et al., 228 were included in this review [2]. Seven were excluded as they were not published in a peer-reviewed journal (see Table 1). Full details of all extracted data can be obtained from the first author on request.

**Table 1:** Excluded studies

**Reference**	**Author**	**Reason for exclusion**
A 4	Anonymous	Report not published in peer r/v journal
A85	Grimshaw	Report not published in peer r/v journal
A86	Grimshaw	Dissertation
A144	Morrison	Report not published in peer r/v journal
A154	Onion	Dissertation
A208	Thomas	Report not published in peer r/v journal
A221	Watson	Dissertation

The average number of outcome measurement categories used in the included studies was 2.1 (SD = 1.0). Table 2 details the number of outcome measurement categories used in the studies and the actual categories used. Overall, the majority (65%) of studies included measures that covered one or two of the outcome measure categories.

**Table 2:** Categories of outcome measures used

**Number of categories**	**Categories used** ** Number of studies**							**Total**
1	A1	B1						75
	2	73						
2	A1, A2	A1, B1	A1, C	A2, B1	A2, C	B1, B2	B1, C	74
	5	6	1	18	2	10	32	
3	A1, A2, B1	A1, A2, C	A1, B1, C	A2, B1, B2	A2, B1, C	B1, B2, C		55
	19	5	9	6	11	5		
4	A1, A2, B1, B2	A1, A2, B1, C	A1, B1, B2, C	A2, B1, B2, C				21
	2	13	1	5				
5	A1, A2, B1, B2, C							3
	3							

A1: Measures of patient change; A2: Surrogate measures of patient change; B1: Measures of practitioner change; B2: Surrogate measures of practitioner change; C: Organisational or process level change

**Figure 1:**
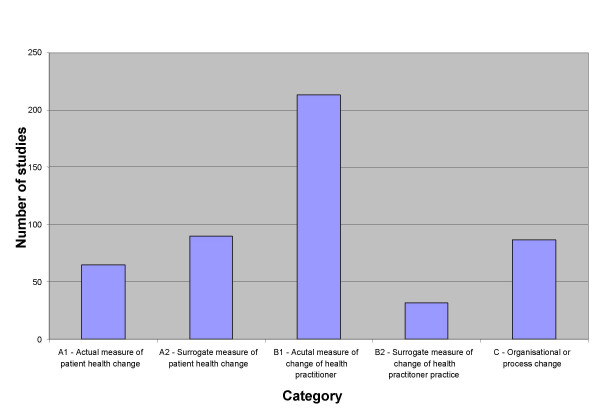
Types of outcome measures used.

Nearly all (93%) of the studies measured outcomes at the level of the practitioner, and 13% used a surrogate measure of practitioner change. Twenty-nine percent of studies used an actual measure of patient change, and 39% used a surrogate measure of patient change. Change was measured at an organisational and/or process level in 38% of studies. (Figure 1)

**Figure 2:**
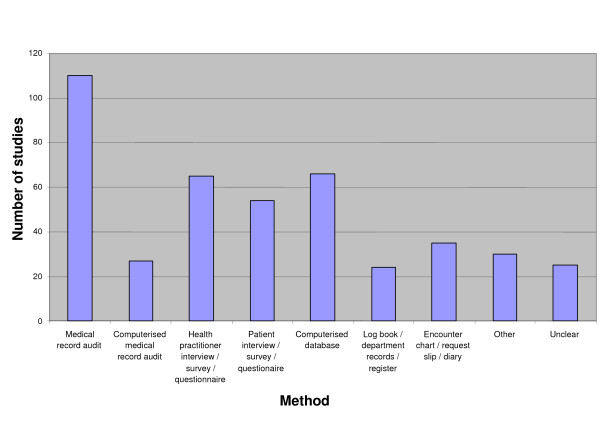
Outcome measure data collection methods.

Twelve studies had unclear methods of data collection and were excluded from the analysis of data collection methods. Approximately one-half (51%, 110/216) of the included studies used a medical record audit to collect data for their chosen outcome. A computerised database was used in 31% of studies, while practitioner interviews, questionnaires and/or surveys were used in 30% of studies. Less frequent was the use of patient interviews, questionnaires, and/or surveys (25%), an encounter chart, request slip or diary (16%), a computerised medical record (13%), and a logbook, department record or register (11%). Other methods were used in 14% of studies. (Figure 2)

Forty-six (20%) of the 228 studies indicated that they had assessed the reliability and/or validity of one or more of their methods used to collect data for their outcome measure(s). Reliability of the medical record audit was assessed in 29 studies. This accounts for 63% of the total number of studies assessing reliability and/or validity, and 26% of the total number of studies that used this method of data collection. Inter- and/or intra-rater reliability of medical record audit was assessed most commonly through a re-audit of a selection of the records.

Thirty-two studies used and referenced at least one previously developed scale and/or questionnaire. In 25 of these studies (78%), the outcome was measured with a patient interview, survey or questionnaire, accounting for almost half (46%) of the studies that measured outcomes with this method. In total, 29 of the studies (91%) that used a previously developed scale and/or questionnaire did so to measure change at the patient level.

### A1: Measures of patient/symptom change

The outcomes in this group measured changes in the patient's health status, either positive (e.g. reduced blood pressure, control of asthma) or negative (e.g. worsening of symptoms, increase in psychosocial problems).

Twenty-six studies used mortality as an outcome measure, while only three studies specifically assessed quality of life. Scales used to assess quality of life included both disease specific and global measures.

The methods used to collect data in this category are summarised in Table 3. The most commonly used method was a patient interview, survey or questionnaire, and disease specific questionnaires were used in ten studies. Other studies using either a patient or practitioner interview/survey/questionnaire tended to use instruments that had been designed specifically for the trial.

**Table 3:** Methods used to collect outcomes for the different outcome measure categories

	**Measure Category**				
	
**Method**	**Measures of Patient Change**	**Surrogate Measures of Patient Change**	**Measures of Practitioner Change**	**Surrogate Measures of Practitioner Change**	**Organisational or Process Level Change**
Medical record audit	32%	29%	45%	0	5%
Computerised medical record audit	14%	9%	12%	0	1%
Medical practitioner interview/survey/questionnaire	12%	2%	5%	100%	53%
Patient interview/survey/questionnaire	37%	4%	10%	0	5%
Computerised database	18%	26%	25%	0	18%
Log book/Department record/Register	2%	7%	9%	0	6%
Encounter form/Request slip/Diary	8%	3%	13%	0	13%
Other	14%	7%	7%	0	8%
Unclear	9%	14%	7%	0	6%

All numbers are percent of the total number of studies for that category

* Excludes 25 studies that measured cost in this category for which no method was recorded

Measurements of actual patient/symptom change accounted for 30% of those in the "other" category of data collection. This reflects the use of clinical tests such as the assessment of smoking cessation through carbon monoxide assessment and/or clinical examination.

### A2: Surrogate measures of patient change

Surrogate measures of patient change were further divided into seven main categories. The methods used to collect data in this category are summarised in Table 3.

Sixteen of the 90 studies assessed patient satisfaction, however, only one of these used a scale that had been documented previously. Where details were provided, the majority of the other studies used questionnaires in which the patients were required to rate on an ordinal scale their satisfaction with various items related to the care they received.

Length of hospital stay was measured in 24 studies, while the number of health care visits and/or hospitalisations was measured in 30 studies, and test (e.g. laboratory) use was measured in eight studies. This information was obtained most frequently through hospital/computerised databases, medical record audit, and/or patient interview.

Ten studies measured patient knowledge and/or attitudes. In six studies, this was assessed through an interview with the patient, and, in the remaining four studies, a questionnaire was used. One trial used a previously developed questionnaire, and three studies used adaptations of previously reported/developed questionnaires. Only three studies reported the results of validity and/or reliability assessments of their tool.

Functional status and/or return to work were assessed in ten studies. A patient interview/questionnaire was used to measure change in this outcome in all of the studies, and all but one of these studies used a previously developed scale/questionnaire.

Patient compliance with treatment/recommendations was assessed in 38 studies, and in 24 of these studies, the intervention was targeted at both the patient and the physician. Therefore, the outcome measure was an assessment of both a surrogate measure of patient change and an actual measure of practitioner behaviour change. In the remaining 14 studies, the measure was purely a reflection of the patient's compliance, i.e., a questionnaire/interview to establish compliance with medical recommendations or compliance with the administration of medications.

### B1: Measures of practitioner change

This category objectively measured a change in the behaviour of the practitioner targeted by the intervention. The primary aim of the measure was not to assess change at the patient level due to a change in the behaviour of the practitioner, rather, the measures used in this category reflected practitioner compliance (or non-compliance) with the implemented guideline.

Measures of practitioner change were the most commonly used outcomes, with 213 of the 228 studies using at least one practitioner change measure. Practitioner change was the outcome measure used in 97% of the studies that measured outcomes in just one category.

Practice change was measured most frequently by medical record audit, computerised databases, encounter forms, request slips or diaries (Table 3). Of the 11 studies using a practitioner interview or questionnaire, two studies referenced assessments of the reliability of their questionnaires, and one study also used an evaluator posing as a patient to check the accuracy of the practitioners' self-reported behaviour. In six of the 11 studies, practitioner interview or questionnaire was the sole means of assessing change at this level.

Twenty-one studies used patient reporting (primarily through an interview) to assess practitioners' compliance. One study assessed the reliability of the patient interview by checking concordance with physician reporting for a random selection of physicians and patients, and one trial used a previously reported scale.

In one study, video-recorded consultations were used to assess practitioner compliance, and two studies audio-taped consultations, one of which reported assessing the reliability of the coding of the taped consultations.

### B2: Surrogate measures of practitioner change

Surrogate measures of practitioner change generally involved the measurement of knowledge and/or attitudes. All outcomes in this category were assessed through a practitioner questionnaire or interview, with the majority using an interview.

Eight studies assessed knowledge alone and seven studies assessed attitudes alone, while 15 studies assessed both knowledge and attitudes. Assessment of attitude in this category encompassed measures that assessed the attitudes of the medical practitioner toward the information being implemented (i.e., attitudes toward preventive medicine), and not toward the method of implementation. Where the trial provided information regarding the assessment of knowledge, the methods used included case scenarios, short answer questions, and multiple-choice questions. Attitudes were most commonly assessed with the use of a Likert scale[8].

Four studies reported using a previously reported questionnaire (or modification of), and seven studies assessed the reliability, feasibility and/or validity of their questionnaire.

### C: Organisational or process level change

The majority of studies assessing outcomes in this category measured cost and/or items relating to the intervention itself.

Cost was the most commonly used outcome measure in this category with 49 (56%) of the 87 studies using this as a measure of organisational change. The use of cost in these studies has been extensively described in the review by Grimshaw et al [2]. The number of studies reported in this review that included cost as an outcome is less than the 63 as reported by Grimshaw et al. [2]. This is most likely due to the inclusion criteria for this review (to be included, the outcome measure needed to be described in the methods and/or results sections of the paper) and to the small number of studies excluded from this review.

No methods were recorded for the collection of costs for 35 studies. In the eight studies that specifically collected data related to cost, a computerised database was used most frequently to obtain the information.

The implementation strategy was assessed in 43 studies. The most common methods for measuring the implementation strategy were through compliance of the practitioners in either implementing the changes or completing the forms, the exposure of the patients and/or practitioners to the interventions and the heath practitioners' acceptance of the strategies, including their perceived usefulness of the strategy. The most common method used to collect this information was a survey or interview of the practitioner (29 studies). An encounter form, chart or diary was the next most common method, with seven studies using this method.

Other measures used in this category included changes in policies and procedures, as well as changes at an organisational level, such as the presence of the required equipment and the impact of the intervention on the time spent by the medical practitioner for each consultation.

## Discussion

While few of the guidelines implemented in the reviewed studies were overtly evidence-based, a large proportion of studies (93%) aimed at improving dissemination and implementation of clinical guidelines by measuring change at the health practitioner level. Less than one-third of studies directly assessed outcomes related to a change in the health of the patient.

When selecting outcome measures for use in studies of interventions to implement evidence and change clinical practice, researchers face the decision of whether to limit assessment to measures of practice (Did the intervention change practice?), or the ultimate endpoint of actual change in patient status or health outcome (Did the intervention improve health status?). When the implementation intervention targets a clinical behaviour for which there is strong evidence of benefit, it may be appropriate to measure outcome only in terms of whether the behaviour occurred, making practitioner behaviour an indicator of the outcome measure or endpoint. In such cases, measurements at the level of the patient, with the associated additional investment and responder burden, may be a waste of resources. However, to fail to do so precludes the study from addressing the overarching question of whether the implementation of evidence results in improved patient outcomes. By documenting the outcome measures used in similar studies to date, we hope to inform discussion and thought regarding the level at which outcome should be assessed in future work.

Regardless of whether measures of practitioner behaviour are viewed as an interim process variable to explain the impact of the intervention on the ultimate endpoint of patient outcomes, or as the primary endpoint of the trial, studies need a valid method of assessing practice change.

Many of the studies did not report the reliability or validity of the methods used to collect data for their outcome measure(s), regardless of the level at which they were assessing change. This invites the possibility that measurement error may bias the results of the investigation of the strategy. The results presented in this review may underestimate the actual number of studies that performed reliability and/or validity testing of their methods, as we were reliant on this being reported in the paper. However, it is likely that the proportion of studies in which reliability of outcome measure and method were determined but not reported is small, and therefore of minimal impact on our results.

It is likely that use of surrogate measures of practitioner change (level B2) is much more prevalent than our study concludes. Primary studies using only this level of measurement were excluded from the review by Grimshaw et al. and, as a result, would not have been captured in our sample, which included this level only if it was used in combination with outcome assessment at another level.

The use of a medical record audit was the most common data collection method. The validity of this method of data collection has been found to be variable depending on the type of information being extracted [9,10]. Approximately one-quarter of the studies using medical record audit attempted to assess the reliability and/or validity of the extraction methods used. In most instances, the testing was limited to assessment of inter-rater reliability. When medical record audits are used, attention needs to be paid to the reliability of the record itself, as well as to the validity of the record and the data extracted from it [11].

Patient and health practitioner questionnaires, surveys, and interviews were also widely used. Measurements of change in practitioner behaviour, knowledge, and/or attitudes (the primary outcome for many studies) through questionnaires, surveys or interviews demonstrated little use of previously developed instruments and poor reporting of reliability and/or validity of the measures used. Those studies assessing patient-related outcomes often used disease-specific or global questionnaires that had been described previously in the literature. The use of patient questionnaires has been shown to have similar variability in validity as that described for medical record audits [10].

Outcome measures used in other areas of healthcare research have been described in similar reviews to our study, i.e., measures used in stroke and shoulder pain trials [12,13]. Others have proposed the use of standard sets of outcome measures in specific areas, such as those recommended for low back pain research [14]. Standardising the use of outcome measures facilitates comparisons between similar studies and pooling of data for meta-analysis. However, unlike studies delivered in defined clinical areas, studies of implementation strategies will need to vary to match the clinical setting, and so a core set of outcome measures may not be possible. However, a common consensus methodology for outcome assessment in studies of implementation may result in improved quality. Such a methodology should include always measuring outcome at least at the level of actual health practitioner behaviour (B1), including measures of patient change where there is not strong evidence to support that the change in behaviour leads to improved patient outcomes, as well as reporting the reliability and validity of data collection methods employed in the study.

## Conclusion

This paper has described the outcome measures used in 228 studies of effectiveness of dissemination and implementation interventions for clinical guidelines. Most trials reported change at the health practitioner level, with less than one-third of trials measuring whether any change in practice resulted in a change in the ultimate endpoint of the patient's health status. Costs were the most reported measure of change at an organisational level. Medical record audit, computerised databases, and health practitioner questionnaire/interview were common ways of collecting data. Very few studies demonstrated the reliability and validity of the methods used. The development of a common methodology for outcome assessment in studies of implementation would facilitate comparisons between studies and the pooling of results.

## Competing interests

Sally Green is Director of the Australasian Cochrane Centre, funded by the Australian Department of Health and Ageing and supported by Monash University. She is a member of the Cochrane Collaboration Steering Group. The views expressed in the present paper represent those of the authors and are not necessarily the views or the official policy of the Cochrane Collaboration (unless otherwise stated and referenced).

## Authors' contributions

SH participated in the development of the methodology, performed the data extraction and primary analysis, and drafted the manuscript. SG conceived the study, participated in the development of the methodology, and helped to draft the manuscript. Both authors read and approved the manuscript.
